# Functional-Group Effect of Ligand Molecules on the Aggregation of Gold Nanoparticles: A Molecular Dynamics Simulation Study

**DOI:** 10.1021/acs.jpcb.2c01132

**Published:** 2022-07-15

**Authors:** Ayse Cetin, Mine Ilk Capar

**Affiliations:** Department of Physics, Faculty of Science, Ege University, Bornova, Izmir 35100, Turkey

## Abstract

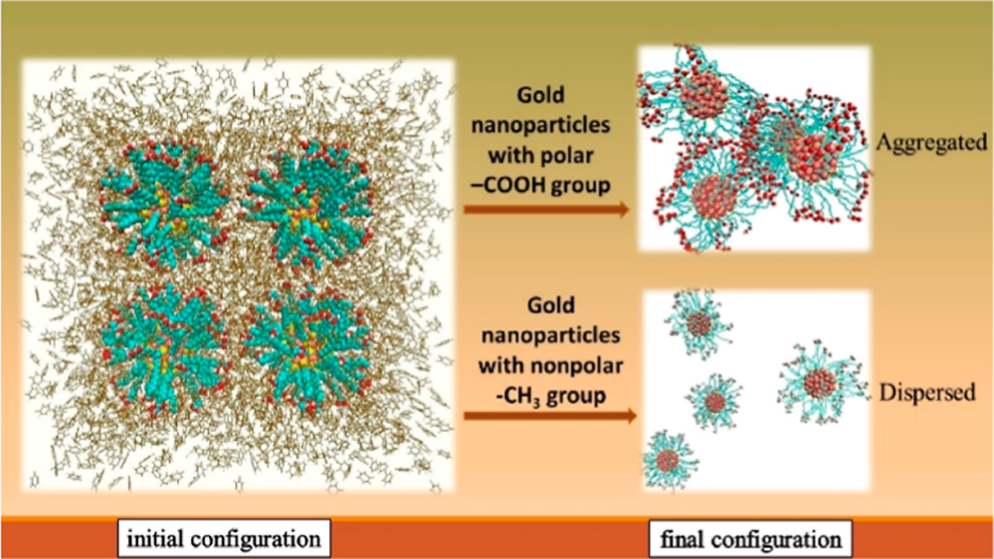

In this paper, atomistic molecular dynamics simulations are performed for the systems consisting of functionalized gold nanoparticles (NPs) in a toluene medium. Gold NPs are coated with ligand molecules that have different terminal groups, that is, polar carboxyl (COOH), hydroxyl (OH), amine (NH_2_), and nonpolar methyl (CH_3_). These functional groups are selected to understand the relation between the aggregation behavior of functionalized gold NPs in toluene and the polarity of terminal groups of ligand molecules. The center-of-mass distances between NP pairs, the radial distribution functions, the mean square displacements, the radius of gyration, and the number of hydrogen bonds (H-bond) between ligand molecules are computed. Our simulation results indicate that functionalized gold NPs exhibit different aggregation/dispersion behaviors depending upon the terminal group of ligands.

## 1 Introduction

Metal nanoparticles (NPs) have several unique physical and chemical properties that cannot be observed in the bulk form of the same materials.^1,2^ Gold NPs have become prominent among metal NPs in recent years due to their chemical stability and nontoxic nature. Their ability to be synthesized in various shapes and sizes and their large surface area–volume ratio result in the gold NPs having unique electronic and optical properties. Because of the significant properties of the gold NPs, they are widely used in various fields of application such as sensors, catalysis, drug delivery, photothermal therapy, and biomedical imaging.^3−8^ The large surface-area-to-volume ratio of the gold NPs allows us to make surface modifications by coating ligand molecules with different functional groups such as hydrophobic, hydrophilic, cationic, anionic, or neutral groups, thiols, DNA molecules, enzymes, and functional polymers.^9−14^ By making surface modifications on the gold NPs, their selectivity to certain surfaces can be improved. The penetration of the particles to the cell membrane is enabled, and they can also carry drugs by holding them on their surfaces.

Depending on the application area, the aggregation or the dispersion of the functionalized gold NPs in a solvent is required. The aggregation of functionalized gold NPs can be the preferred behavior for chemical and biological sensors and also for biomedical imaging applications. On the other hand, it is not desirable behavior in some biological applications such as the injection of functionalized gold NPs into the body for drug delivery. Therefore, determining the factors affecting the aggregation/dispersion behaviors of gold NPs and controlling these behaviors are crucial. Many experimental^15−31^ and molecular dynamics (MD) simulation studies^32−51^ have been performed to understand the aggregation/dispersion behaviors of functionalized gold NPs. In these simulation studies, the coarse-grained (CG) model^32−43^ and realistic atomistic model^44−51^ are widely used. There are a few simulation studies that have examined the effect of the polarity of the terminal groups of ligand molecules on the aggregation of functionalized gold NPs.^34,40,46^

Lin et al. have done the CG MD simulations of functionalized gold NPs in water and butane.^34^ They examined the effects of ligand terminal chemistry, ligand length, solvents, and temperature. In the water medium, they observed less aggregation behavior with increasing polarity of the functional groups. When butane was used instead of water as an environment, it was observed that gold NPs with alkane terminal groups did not aggregate, while gold NPs with polar terminal groups did. Sridhar et al. performed a CG MD simulation study to understand the effect of surface coverage density and chemistry on the self-assembly of monolayer-protected four gold NPs in water solvents.^40^ In addition, the effects of the polar hydroxyl and nonpolar methyl terminal groups of ligand molecules on the aggregation behavior of functionalized gold NPs in the presence and absence of water were investigated by using atomistic MD simulation by Devi.^46^

According to the literature review, most of the MD simulation studies on understanding the aggregation/dispersion behavior of functionalized gold NPs have been focused on the water medium except for a few studies on butane or ethane media.^34,35^ The solvent plays an important role in the aggregation/dispersion process of gold NPs because the interaction between the solvent molecules and ligand molecules affects gold NPs’ behaviors and final morphologies. In order to obtain the desired aggregation or dispersion behavior depending on the application area, the appropriate coating and solvent must be selected. From this point of view, we have chosen nonpolar liquid toluene as an environment, which has not been studied before. The toluene solvent is most frequently used as a nonpolar aromatic compound in synthetic organic chemistry and in industrial processes. In this paper, the effect of the polarity of terminal groups of ligand molecules on the aggregation/dispersion of functionalized gold NPs has been investigated in a toluene medium by using atomistic MD simulation. The gold NPs are coated with polar COOH-, OH-, NH_2_-, and nonpolar CH_3_-terminated alkanethiol ligand molecules. To the best of our knowledge, this is the first MD simulation study in which the toluene medium has been used for understanding the aggregation behavior of functionalized gold NPs.

## 2 Methods

In this study, to investigate the aggregation behavior of functionalized gold NPs, atomistic MD simulations are performed using the GROMACS 5.1.4 program.^52^ The gold NPs are coated with the SH(CH_2_)_11_X ligand molecules by using the PACKMOL program.^53^ Different X terminal groups, which are polar carboxyl (COOH), hydroxyl (OH), amine (NH_2_), and nonpolar methyl (CH_3_), are used. Optimized coordinates of the gold NPs with 144 Au atoms are taken from the previous work.^54^ The Au_144_ NP with a diameter of 2 nm has a nearly spherical rhombicosidodecahedron geometry. It has 30 Au atoms on its surface, 60 atoms are in the lower layer, and the remaining 54 atoms are placed in its center. Each surface Au atom makes a covalent bond with the S atoms of two ligand molecules, that is, S–Au–S. Thus, 30 surface gold atoms are coated with 60 alkylthiol ligands. After coating, four replicated functionalized gold NPs are placed in a rectangular box with the dimensions of nearly 11 × 11 × 6 nm^3^. Then, the box is filled with toluene molecules by using GROMACS tools. The numbers of toluene molecules are set in accordance with the experimental density of liquid toluene^55^ at the temperature used in this research. By changing ligand molecules as SH(CH_2_)_11_COOH (11-mercaptoundecanoic acid), SH(CH_2_)_11_OH (11-mercapto-1-undecanol), SH(CH_2_)_11_NH_2_ (11-amino-1-undecanethiol), and SH(CH_2_)_11_CH_3_ (1-dodecanethiol), four different systems having the same initial conditions are prepared. These are named system I, system II, system III, and system IV, respectively. In each system, the total atom number is approximately 48 000.

**Figure 1:**
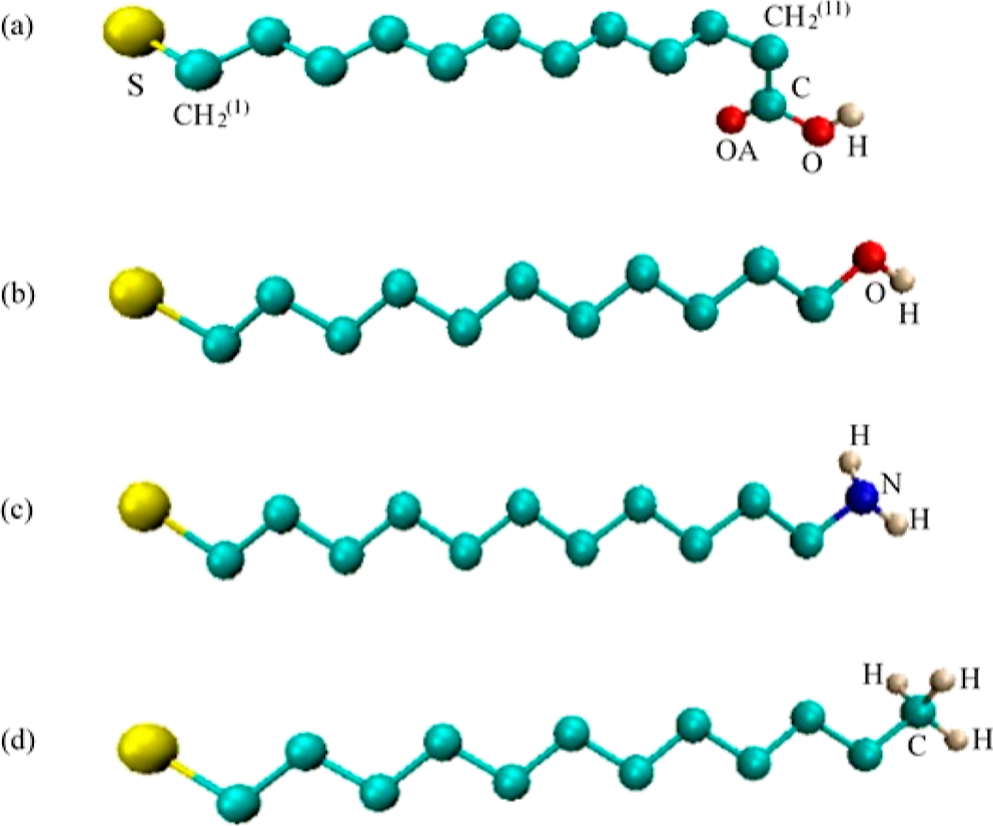
United atom representation of ligand molecules: (a) −S(CH_2_)_11_COOH, (b) −S(CH_2_)_11_OH, (c) −S(CH_2_)_11_NH_2_, and (d) −S(CH_2_)_11_CH_3_. The S atoms are colored yellow, the N atoms are blue, the H atoms are white, the O atoms are red, and the C atoms and CH_2_ groups are turquoise.

**Figure 2:**
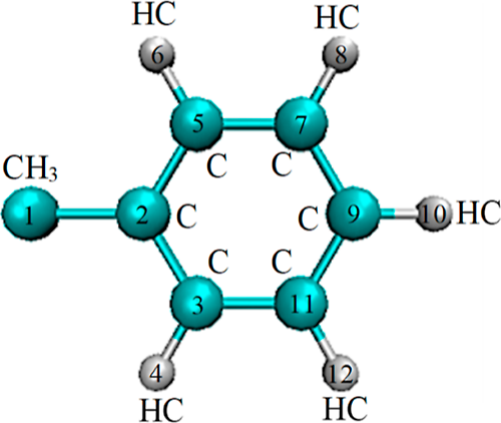
Schematic representation of the model used for toluene molecules.

The united atom model is used for CH_2_ groups in the alkyl chain of ligand molecules and the CH group in the toluene molecule. The schematic representations of ligand molecules and toluene molecules used in this study are given in Figures 1 and 2, respectively. For inter- and intramolecular interactions, the GROMOS force field is used.^56^ The potential function of this force field is given by
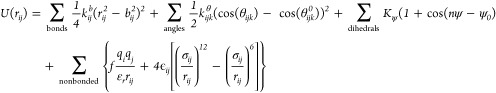
1where the first and second terms, which are defined as the harmonic potential, indicate the bond stretching and angle bending, respectively. The third term is related to dihedral torsion, and the last two terms correspond to the Coulomb and Lennard-Jones (LJ) potentials, respectively. In this equation, *k*_*ij*_^*b*^, *k*_*ijk*_^θ^, and *K*_ψ_ are the force constants, *r*_*ij*_ is the bond length, and *b*_*ij*_ is the reference bond length. θ_*ijk*_ stands for the angle between the atoms, and θ_*ijk*_^0^ is the reference value for this angle. Also, ψ is the angle between the *ijk* and *jkl* planes, ψ_0_ = 0° or 180°, and *n* is the multiplicity of the torsional dihedral angle. In the last two terms in eq 1, *q*_*i*_ and *q*_*j*_ are the charges of atoms *i* and *j*, , and ε_*r*_ is the relative permittivity. ϵ_*ij*_ and σ_*ij*_ are LJ parameters for atoms *i* and *j*; that is, ϵ_*ij*_ represents the depth of the potential well and σ_*ij*_ is the finite distance at which the interatomic potential of *i* and *j* is zero.

The force-field parameters and electrostatic charges are taken from the previous studies for the terminal groups^57−59^ and toluene molecules.^60^ The atomic charges of the Au and S atoms and the CH_2_ groups are taken as zero. All parameters related to the Au and S atoms are taken from the previous study.^54^ The parameters and partial charges belonging to the functional groups in ligand molecules and toluene molecules are listed in the Appendix.

**Table 1:** Time Averages of the COM Distance between Each NP Pair (*d̅*) and the Averages of the *d̅* Values of all NP Pairs () with Standard Deviations

	*d̅*(nm)						
system	1–2	1–3	1–4	2–3	2–4	3–4	*d̅*_pairs_ (nm)
I	3.31 ± 0.03	4.06 ± 0.04	3.34 ± 0.11	2.96 ± 0.04	3.96 ± 0.04	3.99 ± 0.04	**3.60** ± **0.46**
II	3.96 ± 0.05	4.04 ± 0.08	3.32 ± 0.05	3.15 ± 0.05	4.10 ± 0.08	3.49 ± 0.05	**3.68** ± **0.41**
III	4.04 ± 0.09	3.66 ± 0.14	3.96 ± 0.09	4.09 ± 0.08	3.75 ± 0.08	3.87 ± 0.09	**3.90** ± **0.17**
IV	5.66 ± 0.27	8.49 ± 0.42	6.63 ± 0.33	4.95 ± 0.18	7.44 ± 0.38	6.85 ± 0.14	**6.67** ± **1.26**

**Table 2:** Number of H-Bonds between Ligand Molecules of NP Pairs (*n*_HB_)

system	*n*_HB(ligand–ligand)_
I	96.18 ± 6.54
II	93.80 ± 4.75
III	44.23 ± 4.79
IV	

**Table 3:** Diffusion Coefficients of Functionalized Gold NPs

system	*D* × 10^–7^ cm^2^/s
**I**	2.06 ± 0.24
**II**	2.14 ± 0.14
**III**	2.27 ± 0.20
**IV**	3.72 ± 0.48

The steepest descent method is used for energy minimization of the systems. The initial system temperature of 2 K is increased to the target temperature by a heating rate of 0.03 K/ps. The temperature is kept constant at 300 K by a v-rescale thermostat with a time constant of 0.1 ps. The pressure is maintained at 1 bar using a Berendsen barostat with a time constant of 0.5 ps and a compressibility of 4.0 × 10^–5^ bar^–1^ with isotropic coupling. All simulations are performed in the isobaric–isothermal (*NPT*) ensemble with a 0.5 fs time step by using the leapfrog algorithm. The particle mesh Ewald method is employed for long-range electrostatic interactions. The cutoff distance for LJ and electrostatic interactions is set as 1 nm, and periodic boundary conditions are applied in all directions.

## 3 Results and Discussion

**Figure 3:**
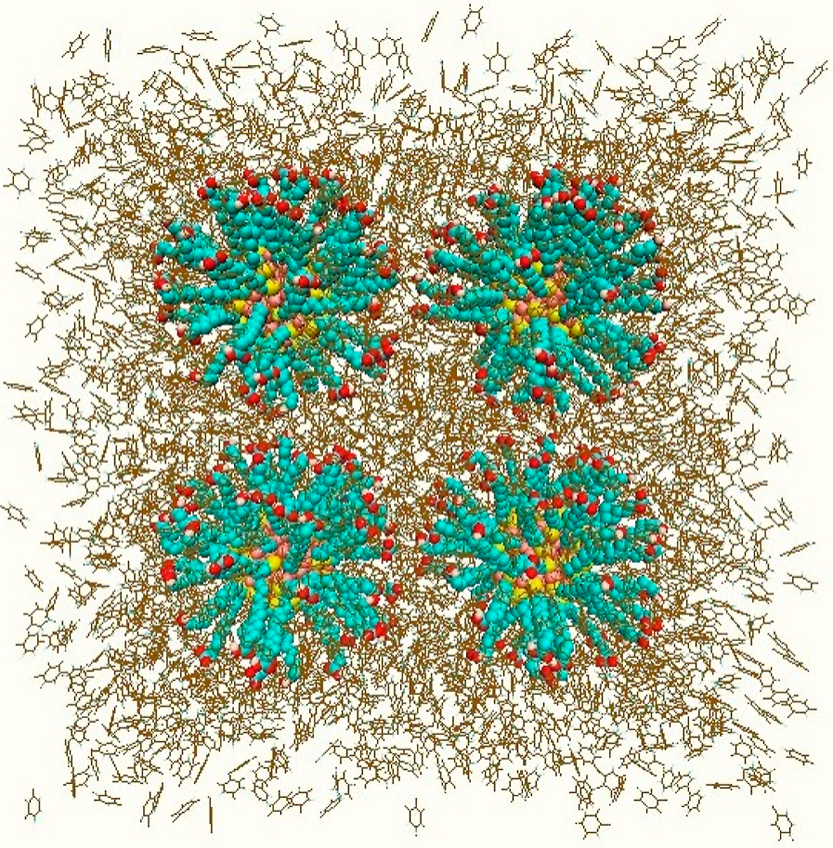
Snapshot of the initial configuration for system II: The gold NPs coated with SH(CH_2_)_11_OH ligand molecules.

**Figure 4:**
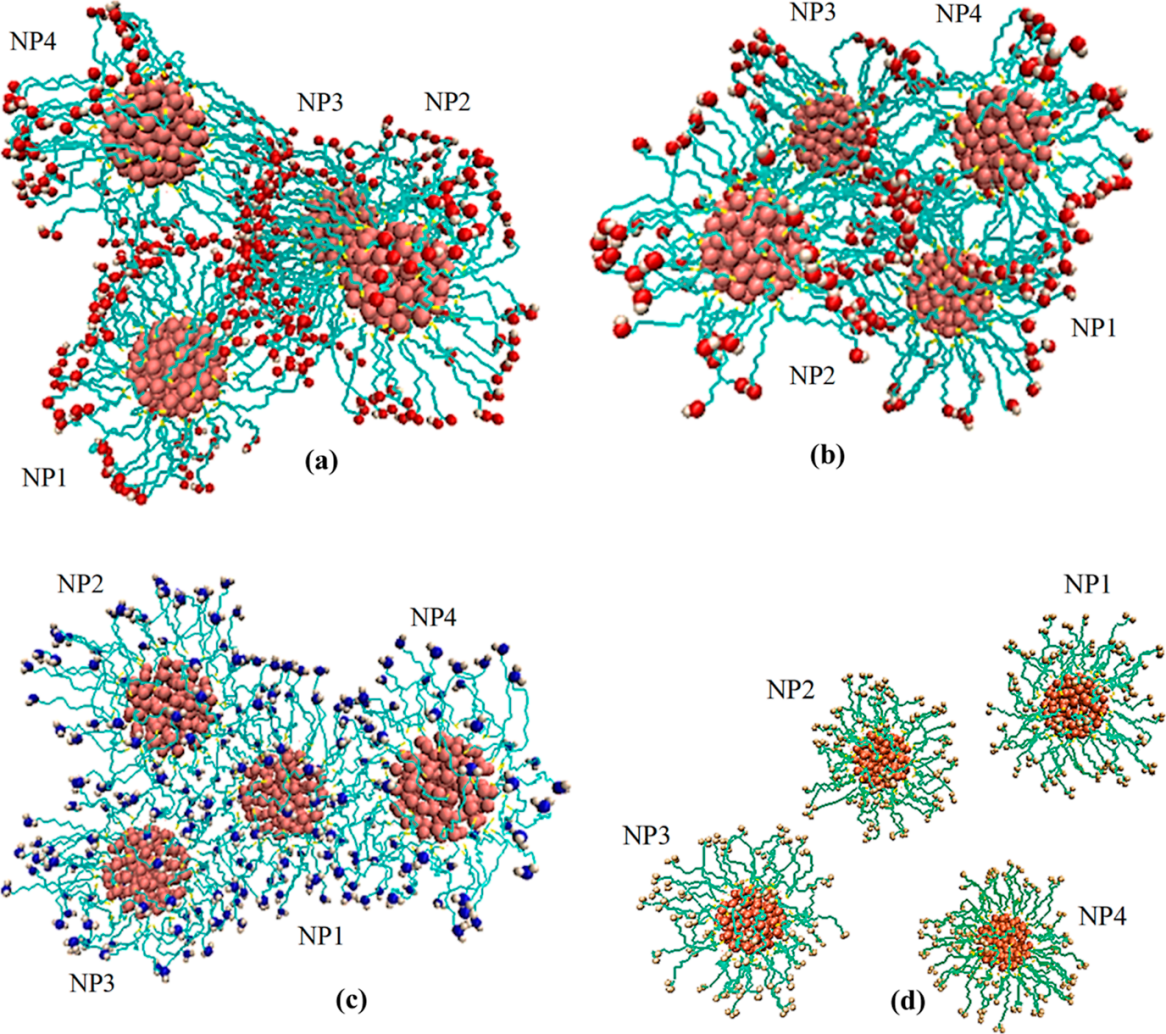
Snapshots of the final configurations of functionalized gold NPs for (a) system I, (b) system II, (c) system III, and (d) system IV. Ligand molecules are SH(CH_2_)_11_COOH, SH(CH_2_)_11_OH, SH(CH_2_)_11_NH_2_, and SH(CH_2_)_11_CH_3_ for these systems, respectively. Alkyl chains are represented as a stick model.

All systems are run for a simulation time of 600 ns. By using the Visual Molecular Dynamics program,^61^ the snapshot of the initial configuration for system II is taken and it is given as an example in Figure 3; the final configurations of functionalized gold NPs for all systems are given in Figure 4. The S atoms are colored yellow, the N atoms are blue, the H atoms are white, the O atoms are red, the C atoms and CH_2_ groups are turquoise, the Au atoms are pink, and the toluene molecules are ochre. After the systems are equilibrated, the calculations are performed for 2 ns simulations by recording MD trajectories more frequently for analyses. The MD simulation trajectories are saved every 20 ps in the production run, and 1000 frames are collected.

To understand the aggregation/dispersion behaviors of the gold NPs coated by ligand molecules with terminal groups of different polarities in the toluene medium, the center-of-mass (COM) distances between NP pairs are calculated. In addition, the radius of gyration, radial distribution functions (RDFs), mean square displacements (MSDs), diffusion coefficients, and number of H-bonds are computed.

**Figure 5:**
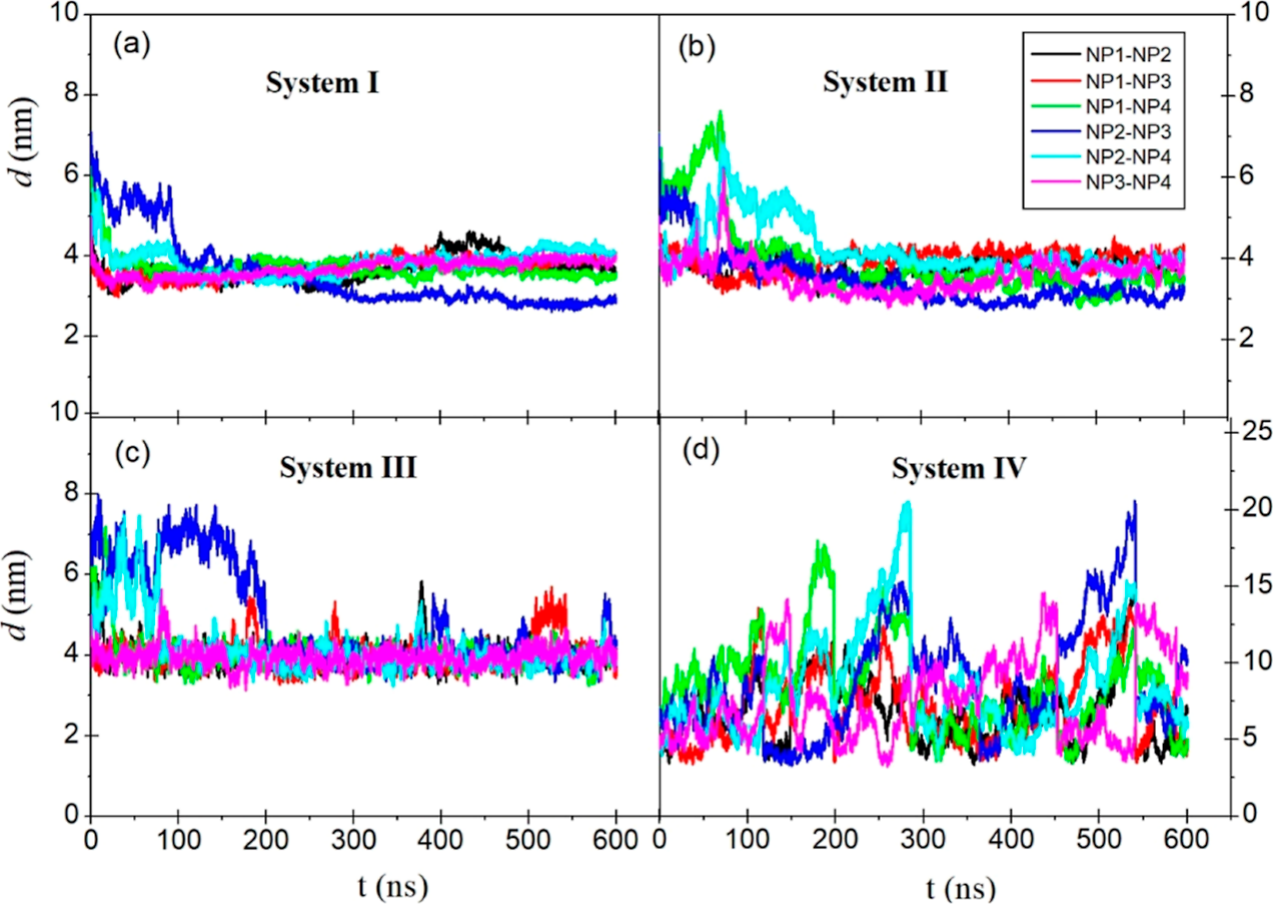
Time evolutions of the COM distances (*d*) between NP pairs.

At the beginning of the simulations (*t* = 0), the positions of NPs are the same for all systems and the average of the COM distance between NP pairs is obtained as approximately 5.68 nm. During the simulation, the COM distance (*d*) between each NP pair is calculated and the time evaluation of *d* values is given in Figure 5. As seen in the figure, the *d* values for systems I, II, and III decrease sharply with time and then remain stable. The decrease in *d* values indicates that the NPs approach each other and remain stable once these systems reach equilibrium. In Figure 5d, the *d* values for system IV take values in a large interval, that is, 5–20 nm. This result indicates that NPs are dispersed.

**Figure 6:**
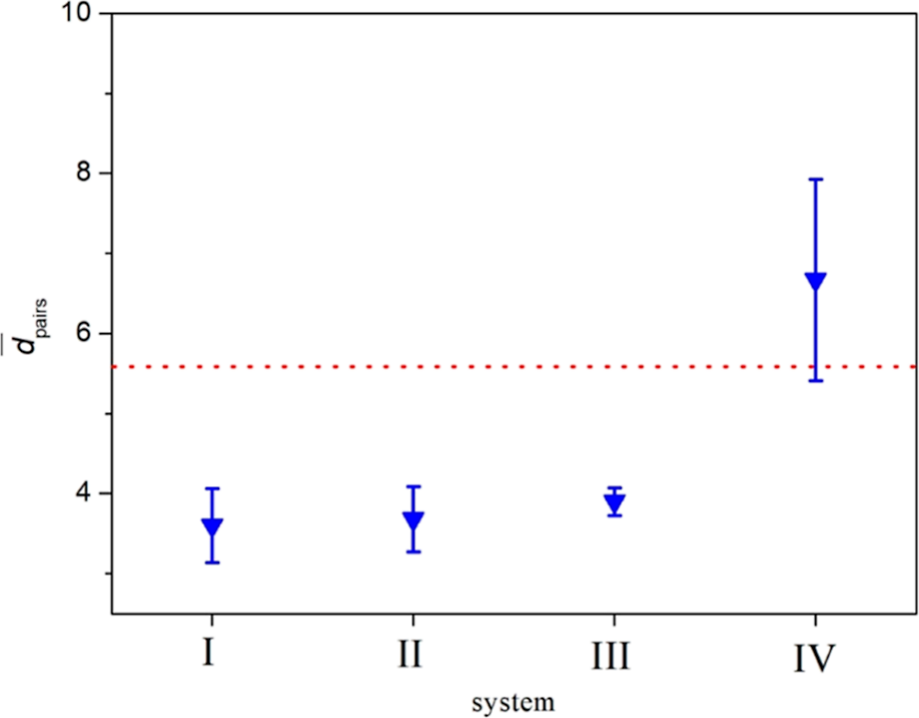
values with standard deviations. The red dotted line shows the average value of the COM distance between NP pairs at the beginning, which is 5.68 nm.

The time average of *d* values over the last 2 ns of the simulation time (*d̅*) for each NP pair and the average of these values over all NP pairs  are given in Table 1.  values for systems I, II, and III are found to be 3.60, 3.68, and 3.90 nm, respectively. All these values are lower than the value of 5.68 nm, which is the average of the COM distance between NP pairs calculated at the beginning of the simulation. The smallest value, 3.60 nm, is found for system I. In this system, gold NPs are coated by the COOH terminal group having the strongest polarity in this study. The  value increases sequentially for system II and system III, while the polarity of the terminal group of these systems decreases gradually. On the other hand, system IV with a nonpolar CH_3_ terminal group has the greatest  value, 6.67 nm. This value is greater than the average of the COM distance between NP pairs at the beginning, 5.68 nm. In order to better understand the size of the aggregation and dispersion among these systems, the  values with standard deviations are given in Figure 6. While the points under the dotted line correspond to the aggregated systems, the upper point is for the dispersed system.

As seen in the snapshots of the last configuration of the gold NPs with the strongest polar group, that is, COOH, the aggregated four NPs form a spherical cluster pattern (Figure 4a). The aggregation pattern looks more planar for the systems with the OH and NH_2_ groups (Figure 4b,c) compared to systems with COOH groups. However, the number of gold NPs in the systems is not large enough to make clear comments about the aggregation patterns. In order to obtain more robust results for the aggregation pattern, the number of gold NPs should be increased.

The hydrogen bonds occur between the polar groups in the surface coverage of each gold NP, and the H-bonds also occur between the polar groups of different gold NPs. The H-bond interactions that occurred between the polar groups of different gold NPs are more important in making a comment about the aggregation behavior of NPs, especially in a nonpolar medium such as toluene. Therefore, in this study, the number of H-bonds between the gold NPs is calculated according to the geometric criteria described in the literature.^62^ The time averages of H-bond numbers between NP pairs and the standard deviations are given in Table 2. The H-bond occurs only between COOH-, OH-, and NH_2_-terminated gold NPs. Therefore, in these systems, the NPs attract each other, and aggregation occurs. For systems I, II, and III, it is observed that the H-bond numbers gradually get smaller by a decrement in the polarity of the functional groups. On the other hand, as a result of the usage of methyl as a functional group, the dispersion of NPs is observed in system IV.

**Figure 7:**
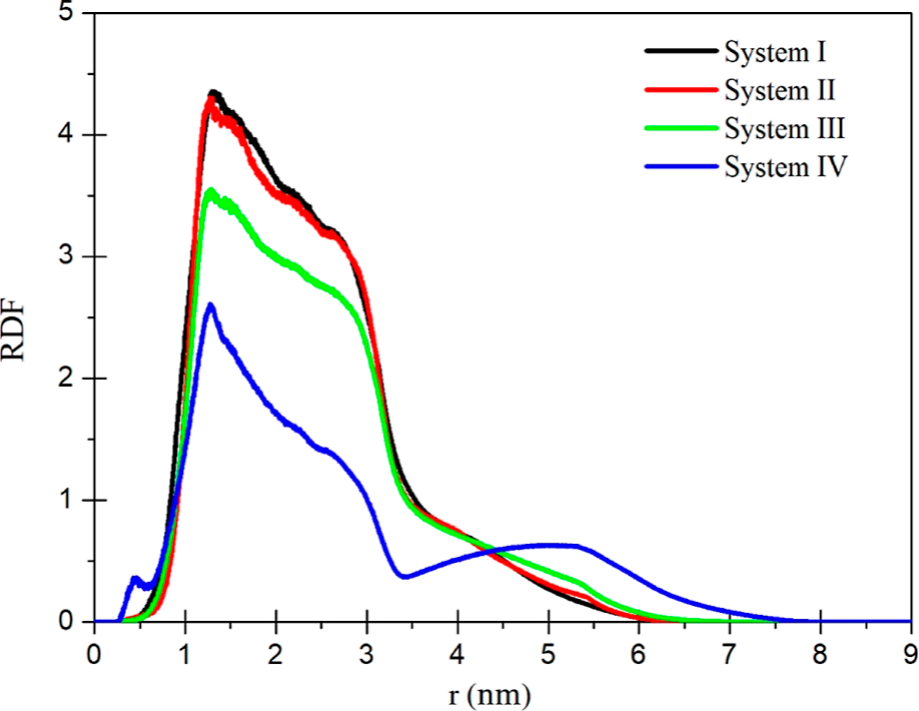
RDFs between the functional group and CH_2_^(1)^ united atom in the ligand molecules.

The RDFs between the functional group and the CH_2_^(1)^ united atom, which is the first atom bonding with the S atom, in the ligand molecules are calculated by using MD trajectory data corresponding to the last 2 ns of the simulation time, and the results for all systems are given in Figure 7. The CH_2_^(1)^ atom is given in Figure 1. As seen in Figure 7, the widths of RDFs for the systems with polar terminal groups are approximately from 0.5 to 6.5 nm. On the other hand, the distribution of system IV with the nonpolar group has a wider width that ends at almost 7.5 nm. While narrow distributions, that is, black, red, and green distributions, for the systems with polar terminal groups are caused by the aggregation of NPs, the wider distribution, namely, the blue distribution, arises from the dispersion of NPs.

The main peak for all systems occurs at nearly 1.4 nm. This value corresponds to the distance between the CH_2_^(1)^ atom and the functional group, which is found by using the optimized coordinates of the ligand molecule from quantum mechanical calculations, and also, this value is almost equal to the length of the ligand molecule. Therefore, it can be said that the chains in the ligand molecules are mostly in the all-trans conformation.

**Figure 8:**
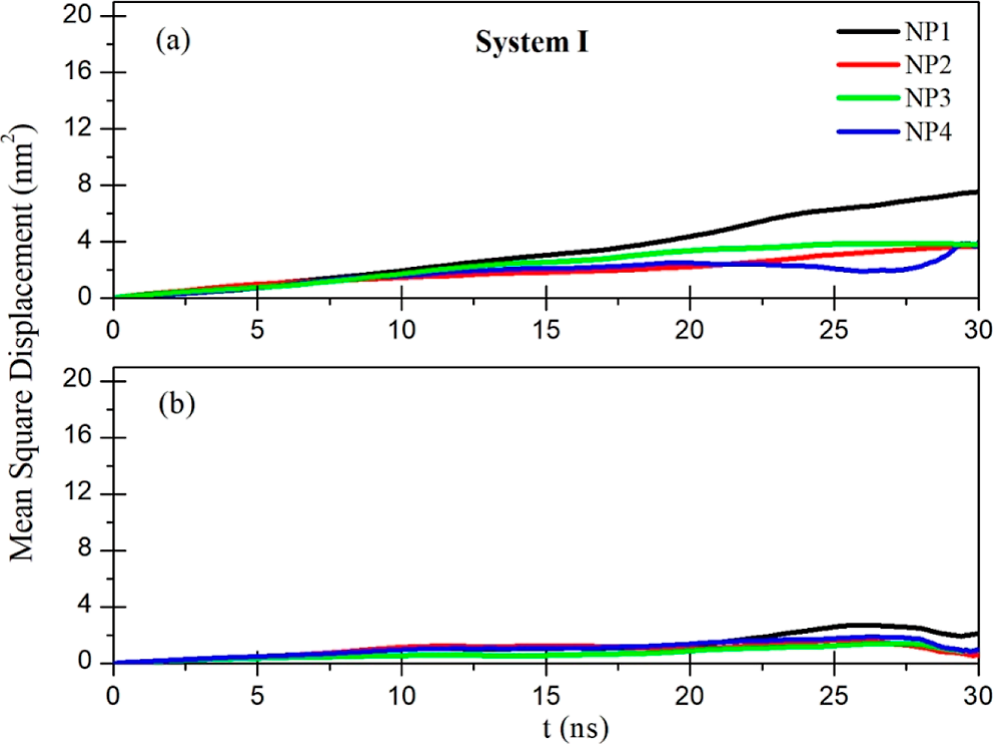
MSDs of functionalized gold NPs with a COOH functional group: (a) first 30 ns and (b) last 30 ns of the simulation time.

**Figure 9:**
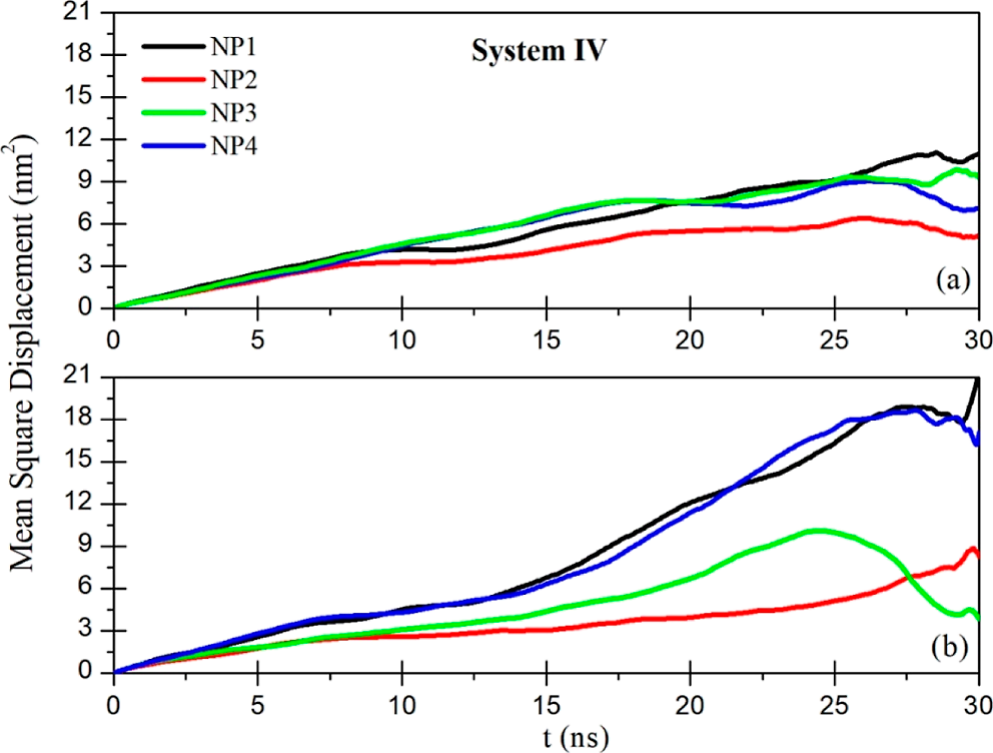
MSDs of functionalized gold NPs with a CH_3_ functional group: (a) first 30 ns and (b) last 30 ns of the simulation time.

To get information about the mobility of functionalized gold NPs in a toluene medium during the first 30 ns (before aggregation/dispersion) and the last 30 ns (after aggregation/dispersion) of the total simulation time, the mean squared displacement of the COM of each NP is calculated and the MSDs for systems I and IV are given as an example in Figures 8 and 9, respectively. The slope of these curves corresponds to the diffusion coefficient (*D*) according to the Einstein relation.^63^ When Figure 8a,b is compared, it can be said that in the last 30 ns period, the MSD curves of NPs for system I with the polar COOH terminal group (Figure 8b) are almost parallel to each other because the NPs aggregate and their trajectories are nearly the same. After aggregation, the slopes of the curves become smaller because the mobility of the gold NPs with a polar terminal group is low.

On the other hand, the MSD curve of system IV with a nonpolar CH_3_ group during the last 30 ns (Figure 9b) has a much greater slope when it is compared with the curves of the systems with polar terminal groups (Figure 8b). This means that the diffusion coefficients of nonpolar functionalized gold NPs are greater than the value of the polar functionalized gold NPs because the NPs in system IV move more freely. The curves in Figure 9b are not parallel to each other because the NPs move away from each other.

The diffusion coefficients for all systems are calculated by using the Einstein relation from the slope of MSD, which is calculated from MD trajectories of 2 ns and given with standard deviations in Table 3.

It can be seen in Table 3 that in the systems with polar terminal groups, the diffusion coefficients of functionalized gold NPs increase with the decrease of the polarity of the terminal groups. The number of H-bonds between terminal groups of the NPs reduces with the decreasing polarity of the terminal groups. When the H-bond number decreases, the mobility of NPs increases, and therefore, the diffusion coefficient increases. The NPs with a nonpolar CH_3_ group can move freely because there is no H-bond interaction between them. Therefore, the highest diffusion coefficient is obtained for this system.

**Figure 10:**
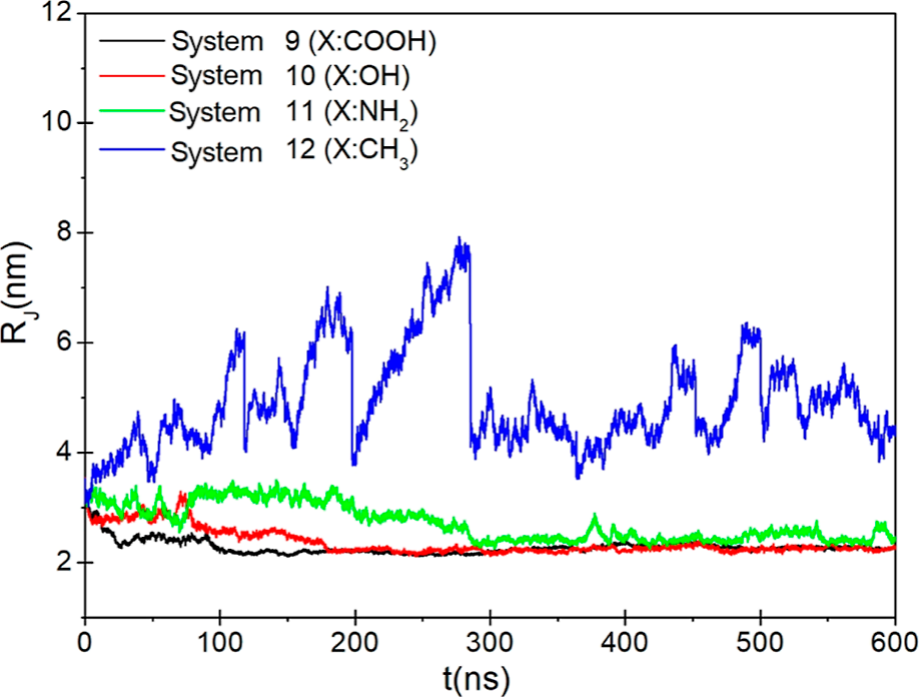
Time evolution of the radius of gyration of gold NPs.

In order to evaluate the size of the aggregate of four functionalized gold NPs, the radius of gyration is calculated by using the following equation
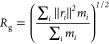
2where *m*_*i*_ is the mass of atom *i* and *r*_*i*_ is the distance of atom *i* from the COM. Figure 10 shows the time evolution of the radius of gyration (*R*_g_). As can be seen in the figure, the initial value of *R*_g_ is almost 3 nm for all systems. While the radius of gyration decreases with the increasing simulation time for the systems with polar terminal groups, it has larger values for the system with a nonpolar methyl group. Smaller *R*_g_ values than the initial value indicate that the NPs get closer to each other; meanwhile, the larger values indicate that the NPs move away from each other.

## 4 Conclusions

In this study, the effect of the polarity of the terminal group on the aggregation of the gold NPs coated with the dodecanethiol ligand molecules in a liquid toluene environment is investigated by using atomistic MD simulation. The terminal group of dodecanethiol ligand molecules is changed to carboxyl, hydroxyl, amine, and methyl groups. The presence of polar functional groups in the ligand molecules of NPs creates attractive interaction contributions at long ranges and causes the H-bonds between the NPs, which are a specific type of dipole–dipole interaction. On the other hand, there is no strong attractive interaction between the NPs with the methyl group because the alkanes are nonpolar, and they interact by means of weak forces. Therefore, the gold NPs with the nonpolar methyl group move independently in the toluene solvent and they do not exhibit aggregation behavior. Calculations indicate that the polar carboxyl-, hydroxyl-, and amine-terminated gold NPs aggregate in the nonpolar toluene solvent, whereas the nonpolar methyl-terminated gold NPs disperse. This result agrees with the previous work^34^ with the butane nonpolar solvent.

Because the electronegativity difference in the COOH, OH, and NH_2_ functional groups gradually decreases, the H-bond interactions between the NPs covered with these functional groups weaken, so the size of aggregation gradually reduces. Consequently, an increment in the polarity of the functional groups causes a decrement in the size of the aggregate of functionalized gold NPs. The smallest size of aggregates is obtained in the system with the carboxyl terminal group. The slopes of the MSD curves show that the diffusion coefficients have small values for the gold NPs with polar terminal groups because their mobilities are limited due to their aggregation. The diffusion coefficients increase with the decrease of the polarity of the terminal groups. On the other hand, the diffusion coefficient has the highest value for the gold NPs with the methyl terminal group because they move more freely. Calculation of the radius of gyration supports the result that the gold NPs with polar terminal groups aggregate and the gold NPs with nonpolar terminal groups disperse.

The obtained aggregation/dispersion behaviors of functionalized gold NPs in this study with the nonpolar toluene solvent are exactly contrary to the behaviors of functionalized gold NPs in previous studies in a polar water solvent.^34,46^ It is found that hydrophobic CH_3_ terminal groups lead to the aggregation of gold NPs in a water medium. On the other hand, the aggregation of gold NPs with polar hydroxyl groups is weakened because of the attractive interactions between solvent molecules and terminal groups.^46^

In this study, due to the nonpolar toluene solvent medium, all gold NPs with a polar functional group attract each other, and as a result of this behavior, the aggregation is strong. If the solvent was polar, the polar functional group in ligand molecules would mostly interact with polar solvent molecules nearest to them. In this scenario, the H-bonds would probably occur mostly between the polar functional groups and polar solvent molecules, so the aggregation would not be strong. To make a more detailed comment on the behavior of these gold NPs in polar solvents, we are planning to investigate their behaviors in water in our future study.

As the results obtained in this study present that the aggregation/dispersion behavior of functionalized gold NPs can be controlled by changing the polarity of terminal groups of coated ligand molecules, they have great application potential in diverse fields that requires a better understanding of these behaviors of functionalized gold NPs. Therefore, these observations are thought to contribute to future studies based on the aggregation/dispersion behavior of functionalized gold NPs in nonpolar liquids.

## Appendix

GROMOS force-field parameters for terminal groups were used in the calculations [see eq 1 and Tables 4 and 5].

**Table 4:** GROMOS Force-Field Parameters for COOH, OH, NH_2_, and CH_3_ Groups in Ligand Molecules

functional group	atom	mass (amu)	*q*(e)	functional group	atom	mass (amu)	*q*(e)
COOH	C	12.011	+0.530	CH_3_	C	12.011	–0.180
	OA	15.999	–0.380		H	1.008	+0.060
	O	15.999	–0.548		H	1.008	+0.060
	H	1.008	0.398		H	1.008	+0.060
OH	CH_2_^(11)^	14.027	+0.150	NH_2_	NT	14.007	–0.830
	O	15.999	–0.548		H	1.008	+0.415
	H	1.008	0.398		H	1.008	+0.415

bond	*r*_eq_ (nm)	*k*_*r*_ (kJmol^–1^ nm^4^) × 10^6^
CH_2_–O	0.143	8.18
C–O	0.136	10.20
C=OA	0.123	16.60
O–H	0.100	15.70
CH_2_–N	0.147	8.71
N–H	0.100	18.70
CH_2_–C	0.153	7.15
C–H	0.109	12.30

angle	θ_eq_ (deg)	*k*_θ_ (kJmol^–1^)
C–O–H	109.5	450.00
CH_2_–CH_2_–O	111	530.00
CH_2_–C=OA	125	750.00
O–C=OA	122	700.00
H–N–H	109.5	380.00
CH_2_–CH_2_–N	111	530.00
CH_2_–N–H	109.5	425.00
CH_2_–C–H	109.5	292.88
H–C–H	107.8	276.14

torsion	Ψ	*C*_*n*_ (kJmol^–1^)	*n*
CH_2_–CH_2_–O–H	0	1.26	3
CH_2_–CH_2_–CH_2_–O	0	5.92	3
CH_2_–CH_2_–C–O	0	1.00	6
CH_2_–CH_2_–C=OA	0	1.00	6
CH_2_–C–O–H	180	16.70	2
OA=C–O–H	180	16.70	2
CH_2_–CH_2_–C–H	0	1.00	6
CH_2_–CH_2_–CH_2_–C	0	5.92	3
CH_2_–CH_2_–N–H	0	3.77	6
CH_2_–CH_2_–CH_2_–N	0	3.77	6
CH_2_–CH_2_–CH_2_–CH_2_	0	5.92	3

**Table 5:** GROMOS Force-Field Parameters for Toluene Molecules

atom type	mass (amu)	*q*(e)
^1^CH_3_	15.035	–0.076
^2^C	12.011	+0.290
^3^C	12.011	–0.260
^4^HC	1.008	0.142
^5^C	12.011	–0.260
^6^HC	1.008	0.142
^7^C	12.011	–0.100
^8^HC	1.008	0.122
^9^C	12.011	–0.157
^10^HC	1.008	0.135
^11^C	12.011	–0.100
^12^HC	1.008	0.122

bond	*r*_eq_ (nm)	*k*_*r*_ (kJmol^–1^ nm^4^) × 10^6^
^1^CH_3_–^2^C	0.152	5.43
^2^C–^3^C	0.140	8.54
^2^C–^5^C	0.140	8.54
^3^C–^4^HC	0.109	12.30
^3^C–^11^C	0.139	8.66
^5^C–^6^HC	0.109	12.30
^5^C–^7^C	0.139	8.66
^7^C–^8^HC	0.109	12.30
^7^C–^9^C	0.139	8.66
^9^C–^10^HC	0.109	12.30
^9^C–^11^C	0.139	8.66
^11^C–^12^HC	0.109	12.30

angle	θ_eq_ (deg)	*k*_θ_ (kJmol^–1^)
^1^CH_3_–^2^C–^3^C	120	560
^1^CH_3_–^2^C–^5^C	120	560
^3^C–^2^C–^5^C	120	560
^2^C–^3^C–^4^HC	120	505
^2^C–^3^C–^11^C	120	560
^4^HC −^3^C–^11^C	120	505
^2^C–^5^C–^6^HC	120	505
^2^C–^5^C–^7^C	120	560
^6^HC–^5^C–^7^C	120	505
^5^C–^7^C–^8^HC	120	505
^5^C–^7^C–^9^C	120	560
^8^HC–^7^C–^9^C	120	505
^7^C–^9^C–^10^HC	120	505
^7^C–^9^C–^11^C	120	560
^10^HC–^9^C–^11^C	120	505
^3^C–^11^C–^9^C	120	560
^3^C–^11^C–^12^HC	120	505
^9^C–^11^C–^12^HC	120	505

torsion	Ψ	*C*_*n*_ (kJmol^–1^)	*n*
^5^C–^2^C–^3^C–^11^C	180	41.8	2
^3^C–^2^C–^5^C–^7^C	180	41.8	2
^2^C–^3^C–^11^C–^9^C	180	41.8	2
^2^C–^5^C–^7^C–^9^C	180	41.8	2
^5^C–^7^C–^9^C–^11^C	180	41.8	2
^7^C–^9^C–^11^C–^3^C	180	41.8	2
